# Mindfulness based relapse prevention (MBRP, Marlatt) in a naturalistic setting for patients with substance use disorder (SUD)

**DOI:** 10.1192/j.eurpsy.2022.2115

**Published:** 2022-09-01

**Authors:** J. Veeser, C. Hanak, A. Rogiers, M. Devos, N. Page

**Affiliations:** 1 Brugmann University Hospital and Enaden addiction center, Brussels, Belgium, Psychiatry, Brussels, Belgium; 2 Brugmann University Hospital, Psychiatry, Brussels, Belgium; 3 Vrije Universiteit Brussel, Faculty Of Medicine, Brussels, Belgium; 4 CHU Brugmann, Psychiatry, Brussels, Belgium; 5 Enaden, Psychiatry, BRUSSELS, Belgium

**Keywords:** mindfulness, addiction, negative effects, MBRP

## Abstract

**Introduction:**

MBRP has become an established treatment in the field of addiction, but implementing the program in an outpatient setting remains a challenge.

**Objectives:**

We investigated the feasibility of MBRP in an naturalistic outpatient setting and the effect of mindfulness on underlying factors of addiction.

**Methods:**

All patients treated between 2015 and 2019 in the MBRP program at Brugmann University Hospital and Addiction Center Enaden were eligible to participate. Patients were asked to fill in a questionnaire about underlying factors of SUD in the domains of pleasure, emotion regulation, stress, relationship with others and relationship with oneself as well as the effect of the completed training on these factors.

**Results:**

Of the 147(74 F) recruited patients; 32 patients completed the questionnaire. The study population differed in terms of substance (mainly alcohol but also cocaine, cannabis, heroine) as well in their aims towards the substance (reduce, stop or maintaining abstinence). Participation of at least 4 of the 8 sessions was 63 % and overall satisfaction of patients was high. We found a positive effect of mindfulness on all of the underlying factors for SUD. Underlying factors of SUD, as well as the effect of mindfulness on these factors showed strong individual variation. The most frequently observed negative effect was acute craving; 1 patient became acute suicidal.

**Conclusions:**

MBRP is feasible and has a clinical relevant impact on underlying factors of SUD. Negative effects were also observed and should be carefully monitored.

**Disclosure:**

No significant relationships.

